# Dual Contraceptive Use and Factor Associated with People Living with HIV/AIDS: A Systematic Review and Meta-Analysis

**DOI:** 10.1155/2021/5440722

**Published:** 2021-08-16

**Authors:** Yibeltal Mesfin, Muche Argaw, Shegaw Geze, Bitew Tefera

**Affiliations:** ^1^Department of Midwifery, College of Medicine and Health Science, Wolkite University, Wolkite, Ethiopia; ^2^Department of Nursing, College of Medicine and Health Science, Wolkite University, Wolkite, Ethiopia

## Abstract

**Introduction:**

A dual contraceptive method is the usage of any modern contraceptive method with male or female condoms which could lower sexually transmitted diseases and unwanted pregnancy. Ethiopian standard utilization of dual contraceptive is low. The hassle is more severe for HIV/AIDS-infected people. Therefore, this review was aimed at assessing dual contraceptive utilization and factor associated with people living with HIV/AIDS in Ethiopia.

**Method:**

International databases (PubMed/MEDLINE, Hinari, Embase, African Journals Online, Scopus, and Google Scholar) and Ethiopian university repository online have been covered in this review. Microsoft Excel was used for extraction, and the Stata 14 software program was used for analysis. We detected the heterogeneity between studies using the Cochran *Q* statistic and *I*^2^ test. Publication bias was assessed by funnel plot and Egger's and Begg's tests.

**Result:**

The overall prevalence of dual contraceptive use among people living with HIV/AIDS was 27.73% (95% CI: 20.26-35.19) in Ethiopia. Discussion with the partner (OR: 3.78, 95% CI: 3.08-4.69), HIV status disclosure to the spouse/partner (OR: 2.810, 95% CI: 2.26-3.48), postdiagnosis counseling (OR: 5.00, 95% CI: 3.71-6.75), schooling in secondary and above education (OR: 3.78, 95% CI: 2.41-5.93), partner involvement in counseling (OR: 2.76, 95% CI: 1.99-3.82), urban residence (OR: 2.84, 95% CI 2.03-3.94), and having no fertility desire (OR: 4.01, 95% CI 2.91-5.57) were significantly associated with dual contraceptive use.

**Conclusion:**

Dual contraceptive utilization among people living with HIV/AIDS was found to be low in Ethiopia. This will be a significant concern unless future intervention focuses on rural residence, involvement of the partner in postdiagnosis counseling, encouraging the people living with HIV/AIDS to disclose HIV status, and discussion with the partner. Providing counseling during the antenatal and postnatal period also enhances dual contraceptive use.

## 1. Background

Human immunodeficiency virus (HIV) is one of the significant pandemics the world faced and ongoing for 40 years [1]. According to the World Health Organization (WHO) 2020 report, about 37.6 million individuals worldwide are infected with HIV, while 1.5 million people become infected for the first time; nevertheless, the reduction is not fast enough to meet the objective of 500,000 people by 2020 [2]. Around 690,000 people died from acquired immunodeficiency disease syndrome- (AIDS-) related illnesses in 2020. Despite the fact that the global new HIV infection reduced by 16 percent across the countries, there were still 6200 new infections each week for young girls between age 15 and 24 groups [2]. In sub-Saharan Africa, four in five new infections are between age 15 and 19 groups [3].

Globally, because of poor dual contraceptive utilization and unsafe sexual practice, over 2 million HIV-infected women become pregnant every year. Women infected with HIV/AIDS have unplanned pregnancies due to lack of access to health service, poor education, sexual violence, or failure of the family planning method [4]. The practices of poor dual contraceptive not only lead to unplanned pregnancies but also increase epidemic of HIV/AIDS and other strains of sexually transmitted infections (STIs) [4–6]. Evidences also showed that more than half of unintended pregnancy occurred among adolescent mothers ending with unsafe abortion [7].

A dual contraceptive method is the utilization of any modern contraceptive method along with male or female condoms which can decrease sexually transmitted disease (STD) and unwanted pregnancy. Consistent and correct use of a condom alone is highly effective for preventing STIs and unwanted pregnancy. However, typical use of a male condom as a contraception method may cause 15% of unwanted pregnancy [8]. Other hormonal contraceptives like combined oral contraceptive, injectable, implants, and intrauterine device protect pregnancy more but do not protect against HIV/AIDS and STIs [8, 9].

Utilization of dual contraceptive among people living with HIV/AIDS (PLWHA) is low in many developing countries, including Ethiopia. Even though a national report was not conducted, studies in various regional states of Ethiopia showed that dual contraceptive utilization among PLWHA is low. For example, in studies from Borena District, Northeast Ethiopia (19.4%), Gondar (13.2%), and Tigray (15.7%), PLWHA used dual contraceptive methods [10–12].

Previous studies revealed that there are different enabling factors for dual contraceptive utilization for PLWHA. Urban residence, disclosing HIV status to a partner, secondary and higher educational level, partner involvement in counseling, postdiagnosis counseling about family planning, age of the women, discussion with the husband/partner, and having a higher CD4 cell count were significant factors affecting the use of dual contraceptive methods [11–13].

Despite the fact that there are publications on the prevalence of dual contraceptive usage and factors associated with PLWHA in Ethiopia's several regional states, there were no studies conducted that indicate the overall level of dual contraceptive use among Ethiopian PLWHA nationally. Therefore, the aim of this review is to have a summary of pooled prevalence dual contraceptive use and factor associated with PLWHA in Ethiopia.

## 2. Methods

### 2.1. Study Design and Search Strategy

We searched studies which reported on the prevalence of dual contraceptive use and associated factor with PLWHA in Ethiopia. This systematic review included studies found in PubMed/MEDLINE, Hinari, Embase, African Journals Online, Scopus, and Google Scholar and Ethiopian university repository online. All published articles as of May 10, 2021, were included in the review. A search of the reference list of the already identified studies to retrieve additional articles was done. The search terms used were “PLWHA dual contraceptive use OR HIV-positive women contraceptive use during ART OR determinants dual contraceptive of use AND Ethiopia”. This was a systematic review and meta-analysis strictly adherent with the Preferred Reporting Items for Systematic Reviews and Meta-Analyses (PRISMA) protocol.

### 2.2. Eligibility Criteria

#### 2.2.1. Inclusion Criteria

All cross-sectional (CS) published and unpublished studies which reported the prevalence and associated factor with PLWHA in Ethiopia with English language research articles were included in the review. Studies were searched from April 1 to 30, 2021.

#### 2.2.2. Exclusion Criteria

Articles whose full text and abstract were unable to be accessed were excluded. Duplicated studies were also checked and removed before the pooled process started.

### 2.3. Data Extraction and Quality Assessment

All articles were extracted and reviewed by two authors (MA and SG) independently with the standard Microsoft Excel spreadsheet form. Disagreement between these authors is handled by discussion with other two authors (YM and BT). The sample size, year of publication, study design, name of authors, region where the study was conducted, prevalence of 95% CI, response rate, and odds ratio were extracted from the original studies.

#### 2.3.1. Outcome Variable

The primary outcome variable of this systematic review and meta-analysis was the prevalence of dual contraceptive use among PLWHA. This was calculated by the pooled prevalence of dual contraceptive use among PLWHA.

The other outcome variable of this review was factors associated with dual contraceptive use among PLWHA in any age group. Calculated by factors associated with dual contraceptive use to each study log, an odds ratio was used.

1. Dual contraceptive use: dual contraceptive use is the utilization of a condom plus any other family planning method by PLWHA for the previous one month
2. Modern methods: the modern methods include sterilization, pills, intrauterine device, injectable, implants, and condom
3. Natural methods: the natural methods include temperature awareness, calendar, cervical mucus, lactational amenorrhoea, and coitus interruptus

#### 2.3.2. Data Processing and Analysis

The Microsoft Excel spreadsheet form was used for extraction, and the Stata 14 software program was used for analysis. The overall pooled prevalence of dual contraceptive use was computed using random effects meta-analysis. Publication bias was assessed by funnel plot through visual assessment. Egger's and Begg's tests at 5% were also performed to detect publication bias. We detected the heterogeneity between studies using the Cochran *Q* statistic and *I*^2^ test. Subgroup analysis by region and year was also calculated to compare dual contraceptive utilization across regions of the country. The point prevalence with a 95% confidence interval (CI) was presented with forest plot format. The relation between the dual contraceptive and the associated factors was determined by the adjusted odds ratio (AOR).

## 3. Result

### 3.1. Description of the Included Study

**Figure 1:**
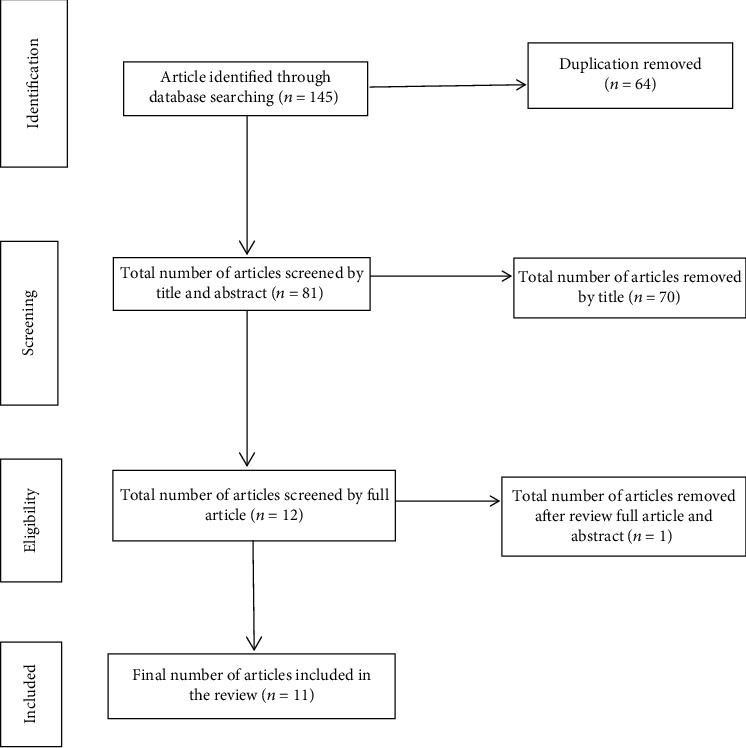
Flow diagram of study selection for systematic and meta-analysis of dual contraceptive use in Ethiopia.

We retrieved 145 studies from the PubMed/MEDLINE, Hinari, Embase, African Journals Online, Scopus, and Google Scholar and Ethiopian university repository online research articles. After the duplicated studies were removed, 81 articles remained. Then, 70 articles were excluded through the reading of their abstract and titles. Thus, 12 full-text articles were assessed for eligibility criteria, further yielding the exclusion of 1 article. Finally, the remaining 11 articles were used to assess the pooled prevalence of dual contraceptive use and its determinant (Figure 1).

### 3.2. Characteristics of Identified Studies

In this review, 11 cross-sectional studies were included. All studies were conducted after 2014. About 4386 study population was included to estimate the pooled prevalence. Five regions were represented for this review. Three from the Amhara region, three from the Oromia region, two from the Southern Nations, Nationalities, and Peoples' Region (SNNPR), two from the Tigray region, and one study from the Benishangul-Gumuz region in Ethiopia were included (Table 1).

### 3.3. Prevalence of Dual Contraceptive Utilization

**Figure 2:**
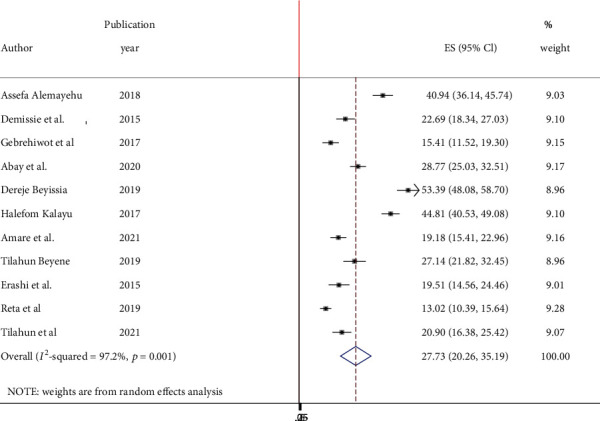
Forest plot in pooled prevalence of dual contraceptive use in Ethiopia.

The lowest prevalence of dual contraceptive utilization was 13% observed in a study conducted in the Amhara region, and the highest prevalence was also 53.4% observed in a study conducted in the Amhara region. The pooled national level of dual contraceptive utilization was 27.73% (95% CI: 20.26%, 35.19%) based on the random effects analysis (Figure 2).

### 3.4. Subgroup Analysis

**Figure 3:**
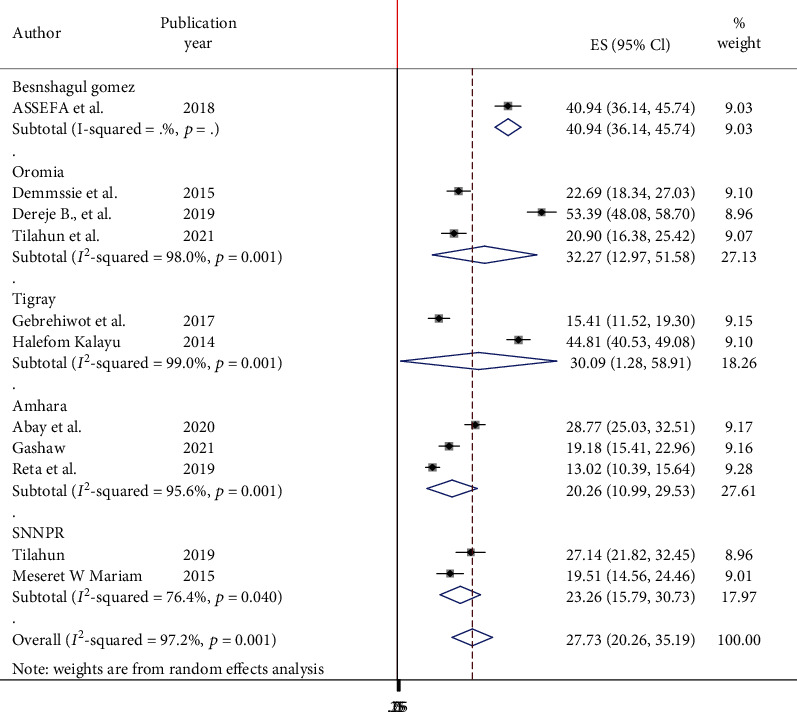
Forest plot showing the subgroup analysis of dual contraceptive in Ethiopia by region.

Subgroup analysis by region and year was also calculated to compare dual contraceptive utilization across regions of the country. Accordingly, the highest dual contraceptive utilization was observed in Benishangul-Gumuz (40.94%, 95% CI: 36.14, 45.74) followed by the Oromia region (32.27%, 95% CI: 12.97, 51.58) and Tigray region (30.09%, 95% CI: 1.28, 58.91), whereas the lowest utilization of dual contraceptive (20.26%, 95% CI: 10.99, 29.53) was observed in the Amhara region (Figure 3).

### 3.5. Heterogeneity and Publication Bias

**Figure 4:**
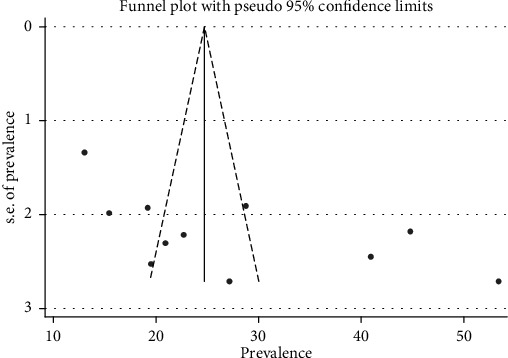
Funnel plot depicting publication bias of studies reporting the dual contraceptive use in Ethiopia.

The *I*^2^ (variation in ES attributable to heterogeneity) test result showed that there was high heterogeneity with *I*^2^ = 97.2, at *p* value < 0.0001. We also assessed the presence of publication bias among studies included in the review. The funnel plot showed that there was an asymmetrical distribution of the included studies through visual inspection, which indicates that there was potential publication bias (Figure 4). However, the Egger regression test (*p* = 0.032) and Begg rank correlation statistics (*p* = 0.161) showed that there was no evidence of potential publication bias.

### 3.6. Sensitivity Analysis

**Figure 5:**
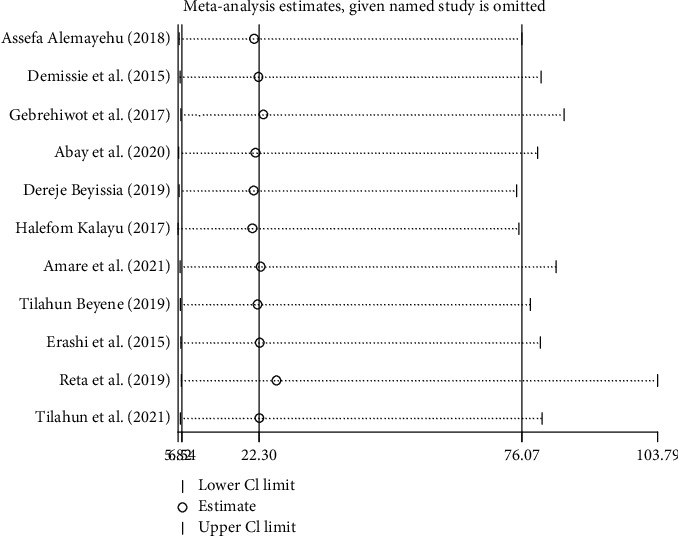
Sensitivity analysis of the prevalence of dual contraceptive use in Ethiopia.

We conducted a sensitivity analysis using a random effects model. Hence, the results showed that no single study influenced the overall pooled proportion of dual contraceptive utilization among PLWHA (Figure 5).

### 3.7. Factors Associated with Dual Contraceptive Utilization

The current review identified different factors associated with dual contraceptive utilization for PLWHA in Ethiopia. Significantly associated factors were residence, disclosure, discussion with the partner about family planning, counseling about family planning, positive partner, fertility desire, and educational status.

The PLWHA residence was significantly associated with dual contraceptive. Using the studies included in the group of meta-analysis, PLWHA who live in urban areas were 2.84 times more likely to use dual contraceptive (AOR: 2.84, 95% CI 2.03, 3.94) than those living in rural residence.

In this review, PLWHA who have discussed with their partner were 3.78 times more likely to use contraceptives (AOR: 3.78, 95% CI: 3.08-4.69) than those who have not discussed with their partner.

Other significant factors associated with dual contraceptive utilization were disclosure of HIV status with the partner. Dual contraceptive use was more likely on PLWHA who disclose their HIV status to their partner than on their counterpart (AOR: 2.810, 95% CI: 2.26-3.48).

Those PLWHA who counseled about family planning ever were 5 times more likely to use dual contraceptives as compared with those not getting any family planning counseling (AOR: 5.00, 95% CI: 3.71-6.75). Similarly, individuals whose partner is involved in counseling were 2.76 times more likely to utilize dual contraceptives as compared with their counterpart (AOR: 2.76, 95% CI: 1.99-3.82).

Educational status is also a novel factor affecting dual contraceptive use. PLWHA who finished secondary and above education were 3.78 times more likely to use dual contraceptives than their counterpart (AOR: 3.78, 95% CI: 2.41-5.93). Additionally, PLWHA who have no desire were 4.01 times more likely to use dual contraceptive than those having the desire in the future (OR = 4.01, 95% CI 2.91, 5.57).

## 4. Discussion

Dual contraceptive uses among PLWHA have an enormous benefit to controlling unwanted pregnancies, decreasing unsafe abortions, and reducing HIV-positive deliveries. So this review was aimed at assessing dual contraceptive utilization and factor associated with people living with HIV/AIDS in Ethiopia. In this meta-analysis, the pooled prevalence of dual contraceptive use among PLWHA in Ethiopia was 27.73% (95% CI: 20.26%, 35.19%). Despite the fact that no comparable meta-analysis study on this specific topic has been conducted in the area, the overall prevalence of dual contraceptive use by PLWHA is analogous to studies conducted in southeast Nigeria (27.7%) [14]; Soweto, South Africa (33%) [15]; Brazil (28%) [16]; and India (23%) [17]. This finding is higher than studies conducted in Uganda (3.5%) [18] and Zambia (17.7%) [19]. But it is lower than studies conducted in Bungoma County, Kenya (38%.5) [20]; Nigeria (45%) [21]; and United States of America (47%) [22]. This difference might be because of regional variation and variation in guidelines of reproductive health and health policy of the country.

This study showed that HIV-positive women who were living in urban areas were more likely to use dual contraceptive methods as compared to those living in rural areas. This is consistent with the finding from Malawi and Togo [23, 24]. The explanation for this might be because of the variation in accessing information and services related to this area. The urban women more easily accessed important information and services related to dual contraceptive utilization through different sources like social media and also from their daily social interpersonal relationships.

In this review, PLWHA who have discussed with their partner were 3.78 times more likely to use dual contraceptives (AOR: 3.78, 95% CI: 3.08-4.69) than those who have not discussed with their partner. This was supported by studies done in Tanzania, Zambia, and Malawi [17, 25, 26]. This is clear that an open communication between couples about sexual and reproductive issue helps to improve contraceptive use. It implies that the need to introduce open discussion between couples on sexual and reproductive issue helps not only to increase dual contraceptive use but also to improve adherence on ART and PMTCT and minimize loss of follow-up for PLWHA [27].

This meta-analysis showed that PLWHA who disclose their HIV statuses to their spouse/partner were found to have significantly increased utilization of dual contraceptives. This finding was supported by studies done in Kenya, Zimbabwe, and Ghana [20, 28, 29]. It is because disclosure facilitating open discussion between partners helps them to mutually accept the importance of dual contraceptive use.

Educational status was also a novel factor affecting dual contraceptive use. People living with HIV/AIDS who finished secondary and above education were 3.78 times more likely to use dual contraceptives than their counterpart. Similar report supports this systematic review from Brazil and Uganda [30, 31]. This can be explained by the fact that educated women have more awareness of dual contraceptive on prevention of HIV/AIDS, unwanted pregnancy, and sexually transmitted disease. Therefore, educating PLWHA is a prominent factor to increase dual contraception use.

PLWHA who had no fertility desire were more likely to use dual contraceptive as compared to their counterpart. This finding is in line with studies from South Africa and Kenya [15, 32]. This may be because couples living with HIV/AIDS who did not want to have a child in the future were more likely use dual contraceptive to protect unwanted pregnancy and STI transmission.

This meta-analysis indicated that PLWHA who received postdiagnosis counseling on family planning were more likely to utilize dual contraceptive methods. The finding is supported by studies from India and Tanzania [17, 25]. This might be explained by the fact that PLWHA have been counseled during their follow-up by a healthcare provider to get more knowledge about reproductive health. Having adequate knowledge about family planning leads to the use of more dual contraceptive to minimize HIV transmission and unintended pregnancy.

Furthermore, the meta-analysis identified that dual contraceptive use was higher in partner involvement in counseling about family planning. The finding was supported by a study from India [17]. It is found in this finding that involvement of the partner in counseling helps the couple for open discussion for their sexual and reproductive health service utilization.

Finally, this systematic review and meta-analysis has limitations. All studies included in this study were cross-sectional, and establishing a cause and effect is imposable due to study design limitation. Additionally, the studies were from five regions only which may restrict the representativeness of the finding.

## 5. Conclusion

The overall utilization of the dual contraceptive method is low. Urban residence, disclosure of HIV status, discussion with the partner about family planning, counseling about family planning, partner involvement in counseling, fertility desire, and educational status were factors affecting utilization of dual contraceptive for PLWHA. Therefore, based on the study findings, husband involvement and providing counseling about contraceptive methods for PLWHA should be encouraged, and there is a need for an intensified effort to improve reproductive health service utilization. Healthcare providers in charge of HIV caregiving must also integrate family planning service counseling in HIV care during follow-up visits.

## Figures and Tables

**Table 1:** Descriptive summary of 11 studies included in the meta-analysis of dual contraceptive use in Ethiopia.

Name of author	Publication year	Region	Study design	Sample size	Response rate
Assefa Alemayehu	2018	Benishangul-Gumuz	CS	403	100
Demissie et al.	2015	Oromia	CS	357	95.2
Gebrehiwot et al.	2017	Tigray	CS	331	97.9
Abay et al.	2020	Amhara	CS	563	0.98
Dereje Beyissia	2019	Oromia	CS	339	94.1
Halefom Kalayu	2017	Tigray	CS	520	99.2
Amare et al.	2021	Amhara	CS	417	98.8
Tilahun Beyene	2019	SNNPR	CS	269	95.9
Erashi et al.	2015	SNNPR	CS	246	98.78
Reta et al.	2019	Amhara	CS	630	98.25
Tilahun et al.	2021	Oromia	CS	311	97.7

## Data Availability

Full datasets and other materials about this study could be obtained from the corresponding author upon reasonable request.

## Abbreviations

AOR: Adjusted odds ratio
AIDS: Acquired immunodeficiency disease syndrome
CS: Cross-sectional
CI: Confidence interval
OR: Odds ratio
HIV: Human immunodeficiency virus
PLWHA: People living with HIV/AIDS
SNNPR: Southern Nations, Nationalities, and Peoples' Region
STIs: Sexually transmitted infections
WHO: World Health Organization.
